# Concomitant presence of culture-proven active pulmonary tuberculosis in patients with chronic obstructive pulmonary disease - A hospital based study

**DOI:** 10.12669/pjms.316.8166

**Published:** 2015

**Authors:** Aneela Liaquat, Shagufta Iram, Shahida Hussain, Noshin Wasim Yusuf, Hassan Azeem

**Affiliations:** 1 Aneela Liaquat, M.Phil Microbiology. Govt. College University Lahore, Pakistan; 2 Shagufta Iram, M.Phil Microbiology, Assistant Professor, Dept. of Pathology, Allama Iqbal Medical College, Lahore, Pakistan; 3 Shahida Hussain, M.Phil Biotechnology, Dept. of Pathology, Allama Iqbal Medical College, Lahore, Pakistan; 4 Noshin Wasim Yusuf, MBBS, MRC.Path, FRC.Path (UK), Professor of Pathology, Rashid Lateef Medical College, Lahore, Pakistan; 5 Hassan Azeem, MBBS, Dept. of Pulmonology. Jinnah Hospital Lahore, Pakistan

**Keywords:** Tuberculosis (TB), *Mycobacteriumtuberculosis* (MTB), Chronic Obstructive Pulmonary Disease (COPD), Pulmonary tuberculosis (PTB), *Ziehl–Neelsen* (ZN), Lowenstein-Jensen(LJ), Mycobacterium Growth Indicator Tube (MGIT)

## Abstract

**Objective:**

To find out the prevalence of concomitant active pulmonary Tuberculosis (TB) in patients of Chronic Obstructive Pulmonary Disease (COPD) using the gold standard liquid and solid culture media for the detection of acid fast bacillus.

**Methods:**

Eighty clinically and radiologically diagnosed cases of COPD of any severity, ≥40 years of age with no previous history of anti-tuberculous therapy were selected from department of Pulmonology, Jinnah Hospital, Lahore. Detailed demographic profile, clinical symptomatology and history of smoking were recorded. Sputum samples of these patients were subjected to ZiehlNeelsen (ZN) stain and culture on Lowenstein-Jensen (L.J) medium and Mycobacterium Growth Indicator Tube (MGIT) for the detection and isolation of *Mycobacteriumtuberculosis* (MTB).

**Results:**

Out of 80 COPD patients, 6 (7.5%) were culture positive for acid fast bacillus consistent with active tuberculous infection. The concomitance was more prevalent in elderly, male, smokers. MGIT was a more sensitive and a rapid technique to detect the presence of mycobacterium as compared to LJ culture media and ZN stain.

**Conclusion:**

The prevalence of active TB in COPD patients was 7.5%. Detection was improved when liquid culture media was employed for the detection of acid fast bacillus. Regular monitoring and screening of patients with COPD for PTB should be routinely carried out in susceptible cohort to avoid cross spreading of infection and appropriate management.

## INTRODUCTION

Chronic Obstructive Pulmonary Disease (COPD) and Pulmonary Tuberculosis (PTB) are widely prevalent respiratory diseases that slowly progress and compromise the lung capacity. The term COPD includes at least three respiratory airways disorders; chronic bronchitis, chronic obstructive asthma and emphysema sharing the common feature of airflow limitation. The patient’s history, physical examination and chest X-ray provide the initial diagnosis of COPD. It is confirmed on Spirometry which is the standard test employed for testing the respiratory function.^1^

COPD affects more than 5 percent of the world population and is associated with high morbidity and even mortality.^2^ It is the third-ranked cause of death in the United States, killing more than 120,000 individuals each year.^3^ An elaborate study conducted in eleven Middle Eastern and African countries reported that around 13 million people in this region suffer from COPD secondary to smoking as the underlying etiological factor. Same study reports a prevalence of 2.1% for COPD in Pakistan.^4^ Another survey reports that 7 million Pakistanis >40 years of age suffer from COPD.^4^ Such high prevalence of this debilitating respiratory disorder puts a heavy burden on social and health sector of our country.

TB is a chronic lung disease caused by the infection by MTB. It remains an important cause of morbidity and mortality worldwide.^5^,^6^ There are 1.3 million estimated deaths annually due to this disease whereas approximately 8.9 to 9.9 million people are newly infected with M. tuberculosis every year.^7^ 95% of these infections occur in the under-developed and developing nations including Pakistan, India, Bangladesh, Afghanistan and Nepal.^8^ Pakistan has the 8^th^ ranking among the countries having the highest burden of TB in the world.

Both TB and COPD remain a major public health issue in our country. Both the diseases share the risk factors like smoking, malnutrition and poor socioeconomic status. It is still not clear if COPD can be a risk factor for TB, independently from smoking. However, in some studies history of PTB is reported to be an independent threat for COPD.^9^-^11^ On the contrary some studies suggest that COPD is not only a sequel of PTB, but may act as a contributory factor for its development.^12^

Occurrence of concomitant TB and COPD is a significant health issue in the TB endemic nations. Inter-relation of these two diseases is complex. The inhalation of smoke may contribute to irreversible airway obstruction in TB endemic regions, even in patients with mild grade of PTB. With healing, PTB can lead to scarring with rapid decline in respiratory function. Also, the obstructive airway changes in treated TB patients increase with age and smoking intensity.^3^,^13^

PTB can also influence the clinical course of COPD. Any past history of PTB augments its progression. Active relapse of TB in such patients can clinically mimic acute exacerbation of COPD.^14^ The COPD patients are not contagious. However in case of co-existing sputum positive active TB they need to be treated immediately to prevent the spread of infection. The co-existence of these two conditions can amplify the ill effects on the patient and the health resources.

The present study was therefore designed to find out the concomitant presence of active TB (culture positive) in population suffering from clinical COPD so that a regular monitoring policy can be recommended.

## METHODS

This is a cross sectional study of a series of 80 clinically, radiologically and spirometrically diagnosed cases of COPD that were selected from the indoor and outpatients of Department of Pulmonology, Jinnah Hospital, Lahore. Patients with hemoptysis, immunospression, on anti-tuberculous therapy or HIV positive status were not included. The demographic profiles, socioeconomic status, smoking status, history regarding the intensity and duration of the symptoms were recorded. The study was approved by the Institutional Ethical Review Board.

Data collected through questionnaire and results of the tests performed were entered into Statistical package for social sciences (SPSS Version 17) and was analyzed descriptively and analytically.

Following the WHO protocol, patients were asked to submit one spot and one morning sputum sample. All the sputum samples were subjected to: AFB smear microscopy by ZN staining and results interpreted according to WHO criteria.

Mycobacterium culture was put up on LJ medium and BACTEC™ MGIT™ 960 system medium after decontamination of sample (according to manufacturer recommended protocol).

Growth of *M. tuberculosis* on LJ media appeared as buffy, granular colonies after 3-7 weeks. Positive MGIT tubes appeared as flocculation, granular and non-homogeneous suspension. The instrument declared a tube negative if it remained negative for 42 Days.

## RESULTS

Eighty patients of COPD admitted in the pulmonology ward of Jinnah Hospital/attending OPD were included in the study. The study population comprised of 74 male and 6 females. Age of the patients ranged between 37-82 years with a mean of 57.9. Only 9 patients out of the study cohort were <50. All belonged to low socioeconomic status. History of smoking was specially probed into regarding duration and frequency. Out of them 83.7% were heavy smokers, 6.3% were moderate smokers and only 2.5%admitted to be mild smokers with one pack/day with 10 years duration. Sputum samples of patients were collected following the WHO protocol. Smears were subjected to ZN staining. Seventy nine patients revealed negative results whereas single patient was ZN positive for AFB (Table-I). All sputum samples were then put up on LJ culture media, the gold standard technique and results recorded after 7 weeks of incubation following the standard protocol. Three cases were positive at various time periods as depicted in Table-II, one of these being the ZN positive case.

**Table-I:** Results of ZN stained smear of sputum samples for AFB (N=80).

ZN stain results	Frequency	Percent	Valid Percent	Cumulative Percent
Negative	79	98.8	98.8	98.8
+ Positive	1	1.3	1.3	100.0
Total	80	100.0	100.0	

**Table-II:** Results of LJ culture of sputum for AFB.

LJ culture	Frequency	Percent	Cumulative Percent
Negative	77	92.8	96.3
LJ culture positive in 22-28 days	1	1.2	97.5
LJ culture positive in 43-49 days	2	2.4	100.0
Total	80	96.4	

Growth of mycobacterium on LJ culture slants. The slants were inoculated with decontaminated sputum sample and were incubated for a period of 8 weeks. The culture reading was done after every week to check the growth of mycobacterium. Out of 80 sputum samples, 1 (1.2%) was positive on LJ culture in 22-28 days (4th week) after inoculation whereas 2 (2.4%) patients came positive on culture in 43-49 days (7th week) after inoculation.

Part of the sputum sample was put up on the MGIT. Six patients out of 80 were positive for AFB. Two cases (2.5%) gave the positive signals in the first week of inoculation whereas, one (1.3%) patient each was found to be positive in 2^nd^, 4^th^, 5^th^ and 6^th^ week(Table-III). Five of these patients were >50 years old, all were males and all 6 were smokers of different grade of severity. We also calculated the sensitivity and specificity of the 3 diagnostic modalities for detecting the acid fast bacillus in sputum samples. Results of MGIT are depicted in Table-IV. The MGIT liquid culture media proved to be the most sensitive and specific for detection of acid fast bacillus.

**Table-III:** Results of MGIT culture of sputum samples (N=80).

MGIT Results	Frequency	Percent	Cumulative Percent
Negative	74	92.5	92.5
MGIT culture positive in 1^st^ week	2	2.5	95.0
MGIT culture positive in 2^nd^ week	1	1.3	96.3
MGIT culture positive in 3^rd^ week	0	0.0	0.0
MGIT culture positive in 4^th^ week	1	1.3	97.5
MGIT culture positive in 5^th^ week	1	1.3	98.8
MGIT culture positive in 6^th^ week	1	1.3	100.0
Total	80	100.0	

Six patients out of 80 were positive for AFB. Two (2.5%) patients were positive in the first week of inoculation whereas, 1 (1.3%) patient each was found to be positive in 2nd, 4th, 5th and 6th week. None was positive in the 3rd week of inoculation.

**Table-IV:** MGIT results versus and LJ culture results.

		LJ Culture (Gold standard)		Total	Sensitivity	Specificity
		Positive	Negative			
MGIT	Positive	3 (TP)	3 (FP)	6	100%	96.1%
	Negative	0 (FN)	74 (TN)	74				
	Total	3	77	80		

(Sensitivity and specificity of MGIT by comparing with Gold standard (LJ)).

## DISCUSSION

The COPD patients run a higher risk of several co-morbidities. These include bacterial infections with relatively resistant strains of pseudomonas and haemophilus influenza. These patients are also at risk for getting infection by MTB with development of active PTB. Among several known risk factors, COPD has also been included as an independent significant risk factor for PTB on multivariate analysis.^15^

Results of the present study show that there is a high risk of TB and COPD co-morbidity (>7%). Several studies have reported even higher figures. Mohapatra et al from India reported concomitant TB in 13.7% of their cases of COPD.^14^ From Sweden, Inghammer et al report that prevalence of active TB in their diagnosed COPD patients is 3 times higher than general population.^16^

The cause-effect relationship of two diseases is complex. Results of meta-analysis carried out by Allwood et al indicate that a significant association exists between past history of PTB and the presence of chronic airway obstruction, independent of smoking.^17^ According to some studies the development of active TB in COPD patients is partly therapy related, attributed to oral corticosteroids.^18^ It has been suggested that deterioration in the clinical status of COPD patients with increase demand for their medication might reflect an underlying active TB infection, thus suggesting a vigorous screening for its concomitant presence.

COPD is a chronic disease that progresses and manifests clinically with advancing age. Our study supports this age related prevalence.85.5% of our study cohort was above 40 years of age whereas 10.8% were <40 years. The risk of concomitant tuberculous infection is also increased in elderly patients. Five of our culture positive patients were ≥50 years of age whereas a single patient was <50 years of age. Our results are concordant with the results of Rizvi et al where COPD and TB co-morbidity was found to be more prevalent in elderly patients (24.3%) as compared to young cases (3%).^19^

The gender association with COPD was recorded in the present study. Prevalence was significantly high in males. The concomitant culture confirmed tuberculosis was also predominantly prevalent in males and all 6 cases positive for AFB on MGIT culture were male subjects. These results depicts that as more male subjects have the tendency to develop COPD, they have an enhanced risk of developing tuberculosis as well as a co morbidity at some stage of their life. Inghammar et al reported similar gender bias for concomitant COPD and tuberculous infection.^12^

Our study explored the relation of history of smoking and its effect on prevalence of COPD and concomitant TB. Incidence of COPD was significantly higher in smokers (92.5% smokers) and all 6 patients who were MGIT culture positive for AFB, were also smokers. Several studies have explored the role of smoking as a contributing etiological factor for PTB. Davies et al in their study reported a 3-5 fold increased risk of developing active TB in smokers.^20^ Inghammar et al in their study also reported that the leading etiological factors like smoking increases the risk of TB infection in patients with COPD.^12^ Considering that active TB in COPD patients can be a source of cross infection and can also increase the need of medications, or simulate the acute exacerbation of illness leading to wrong direction of treatment, its timely detection is of paramount importance. We therefore tried all three diagnostic modalities for detection of active TB infection in our study. The results demonstrated that the MGIT 960 system is much faster and sensitive than LJ media in growing mycobacterium species. It was found to be more accurate and rapid method for the diagnosis of smear positive as well as smear negative TB.^21^ Currently the developing countries rely on ZN-staining for the detection of AFB as it is a simple and cost effective method. However, the present study reveals that despite its feasibility; ZN staining is not a sensitive diagnostic tool for PTB. Due to false negative results a patient with active pulmonary infection can be diagnosed as sputum smear negative. A timely accurate diagnosis can lead to effective treatment of active cases and ultimately help in controlling the high prevalence of disease in our country. Therefore the attempts should be made to improve the diagnostic tools for PTB.

## CONCLUSION

Risk of concomitant COPD and PTB is high in our population. The risk is higher in males and elderly population with history of smoking. Every effort should be made to diagnose presence of active TB in COPD patients using the available sensitive methods of AFB detection. Timely control of this concomitant morbidity will prevent cross infection and deterioration of COPD status.
